# A possible way of sharing experience: encouragement of case reporting from the field

**DOI:** 10.1093/omcr/omab001

**Published:** 2021-03-08

**Authors:** Yoshihiro Aoki, Deirdre J Foley

**Affiliations:** 1 Department of Emergency and Critical Care Medicine, Aizawa Hospital, Nagano, Japan; 2 Department of Paediatrics, Children’s Health Ireland at Crumlin, Dublin 12, Ireland

As discussed by our colleague Wind [1], in a previous letter, we would also like to highlight the importance of sharing knowledge from the field in resource limited settings. As part of our first assignments with Médecins Sans Frontières, we, as pediatricians, participated in fieldwork for a 6-month period each in 2018. The project was based in a conflict setting in northeastern Syria. During this time, we were responsible for overseeing the medical treatment for pediatric patients in both a hospital and community settings. Part of our duties was to provide medical care for a cohort of patients living with thalassemia, an autosomal recessive, transfusion-dependent hemoglobinopathy. There is a baseline high carrier rate of thalassemia among the Syrian population. In this thalassemia clinic, we cared for over 200 patients with transfusion-dependent β-thalassemia. Their essential treatments of safe blood transfusion and appropriate iron chelation therapy had been interrupted due to the conflict. Many patients suffered from complications of iron overload; including cardiac toxicity, delayed growth and puberty and hepatic dysfunction. Although there was inadequate laboratory diagnostics available, some of our patients also displayed symptoms suggestive of hypothyroidism and diabetes mellitus secondary to their complex medical conditions. Furthermore, we observed a high rate of hepatitis C infection and some cases of hepatitis B within our patient cohort, thought to be due to unsafe blood transfusion during the height of the conflict. Although progress was made during our time spent working in the department and significant developments made with patient care guidelines, we feel that there is still room to improve practice to further optimize patient care for this vulnerable cohort in this resource-limited setting. Challenges included limitations in accessing care due to a lack of safe and reliable transportation to thalassemia clinic, as well as complexities in the sociocultural perceptions on HIV testing, the significant psychological morbidity as a result of living in an area of protracted conflict and the provision of care for a complex chronic illness in this resource-limited setting. Documentation of the situation and challenges is necessary to learn and improve practice and to ultimately result in improved patient care and clinical outcomes. We hope that the *Oxford Medical Case Reports* will contribute to increasing the available knowledge base through the submission of papers by medical staff in humanitarian settings sharing their experiences and challenges with the provision care for people living with complex medical conditions in similar resource-limited settings.

## CONFLICT OF INTEREST STATEMENT

None declared.
